# The Influence of Custom-Milled Framework Design for an Implant-Supported Full-Arch Fixed Dental Prosthesis: 3D-FEA Sudy

**DOI:** 10.3390/ijerph17114040

**Published:** 2020-06-05

**Authors:** João Paulo Mendes Tribst, Amanda Maria de Oliveira Dal Piva, Roberto Lo Giudice, Alexandre Luiz Souto Borges, Marco Antonio Bottino, Ettore Epifania, Pietro Ausiello

**Affiliations:** 1 Department of Dental Materials and Prosthodontics at São Paulo State University (Unesp), Institute of Science and Technology, São Paulo 01049-010, Brazil; joao.tribst@unesp.br (J.P.M.T.); amanda.piva@unesp.br (A.M.d.O.D.P.); alexandre.borges@unesp.br (A.L.S.B.); mmbottino@uol.com.br (M.A.B.); 2 Deptartment of Clinical and Experimental Medicine, Messina University, 98100 Messina, Italy; 3 Department of Neurosciences, Reproductive and Odontostomatological Sciences, School of Dentistry, University of Naples Federico II, S, via Sergio Pansini n. 5, 80131 Napoli, Italy; ettore.epifania@unina.it (E.E.); pietro.ausiello@unina.it (P.A.)

**Keywords:** finite elements analysis, computer assisted numerical analyses, mechanical stress, implants prosthetic dentistry, fixed full-arch prosthesis, biomechanics.

## Abstract

The current study aimed to evaluate the mechanical behavior of two different maxillary prosthetic rehabilitations according to the framework design using the Finite Element Analysis. An implant-supported full-arch fixed dental prosthesis was developed using a modeling software. Two conditions were modeled: a conventional casted framework and an experimental prosthesis with customized milled framework. The geometries of bone, prostheses, implants and abutments were modeled. The mechanical properties and friction coefficient for each isotropic and homogeneous material were simulated. A load of 100 N load was applied on the external surface of the prosthesis at 30° and the results were analyzed in terms of von Mises stress, microstrains and displacements. In the experimental design, a decrease of prosthesis displacement, bone strain and stresses in the metallic structures was observed, except for the abutment screw that showed a stress increase of 19.01%. The conventional design exhibited the highest stress values located on the prosthesis framework (29.65 MPa) between the anterior implants, in comparison with the experimental design (13.27 MPa in the same region). An alternative design of a stronger framework with lower stress concentration was reported. The current study represents an important step in the design and analysis of implant-supported full-arch fixed dental prosthesis with limited occlusal vertical dimension.

## 1. Introduction

Implant-supported full-arch dental prosthesis (IFDP) is used as an alternative for total muco-supported prosthesis, achieving appropriate aesthetics, masticatory function and patient satisfaction [1,2]. However, sometimes this treatment cannot be performed due to anatomical and physiological alterations that make it impossible to correctly install the implants and the prosthesis [1]. If possible, bone regeneration and an appropriate prosthetic planning must be performed to avoid future mechanical and biological complications, when the prosthesis is functioning. Chipping, coating material fracture, prosthetic screw loosening and even framework fracture are among the most common mechanical complications [1,2].

The inter-arch distance available for the prosthesis installation represents a great influential factor in the success of the prosthetic rehabilitation. An inadequate inter-arch distance will not allow accommodation of the metal substructure, denture teeth and acrylic resin [1]. Moreover, in the case of a patient having limited inter-arch space, it is necessary to consider the wear of the artificial teeth in resin as any modification in the occlusal vertical dimension. The vertical dimension lost may also complicate future prosthetic approaches, since the resin becomes too thin to accommodate the metallic infrastructure [3].

**Figure 1:**
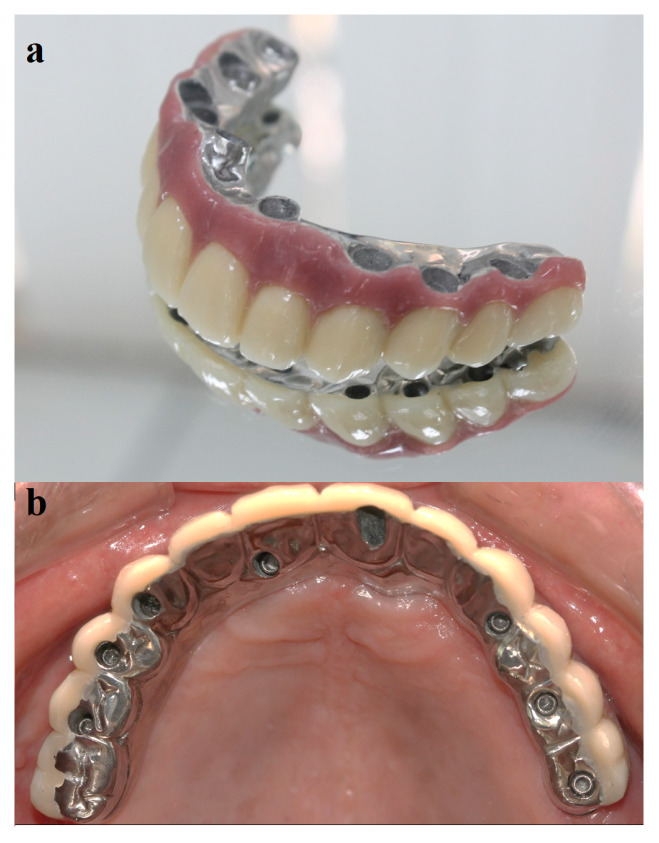
(**a**) Example of experimental prosthesis milled in metal. For this design, only the buccal face receives the esthetic covering. (**b**) An example of occlusal view of the experimental design. This picture has been used as base for the modeling used in the Finite Element Analysis (FEA).

In the case of inadequate inter-arch distance, the design of a milled titanium framework was proposed as an alternative to maintain the occlusal vertical dimension and to prevent the artificial teeth from wear, improving the system mechanical strength (Figure 1). This framework design includes the occlusal and lingual cusps of the posterior teeth as part of the structure [1]. Such an approach, together with a CAD/CAM (computer aided design/computer aided manufacturing) facility, enables the development of a better adapted infrastructure with a lower vertical misfit than the prosthetic abutment [4,5]. To investigate the bone-implant system by means of a three-dimensional (3D) analysis, the Finite Element Analysis (FEA) method is commonly used [6].

It has not yet been demonstrated if there are any mechanical advantages of using a milled framework from a biomechanical point of view. In addition, no longitudinal studies have verified the survival and success of this approach. The current investigation aimed to provide a biomechanical study of different rehabilitations using IFDP. The hypothesis was that the milled framework approach would improve the prosthesis mechanical behavior.

## 2. Materials and Methods

### 2.1. Finite Element Analysis (FEA)

Biomechanical responses in dental applications have been extensively investigated by means of modern CAD–FEM (Computer Aided Design and Finite Element Method) techniques [6,7,8,9,10,11]. In this study, 3D models were investigated by finite element analysis (FEA), according to different prosthesis design. The experimental design consisted of a machined titanium framework with a metallic buccal side (Figure 1).

### 2.2. Generation of FEA Models

The maxillary computed tomography from São Paulo State University database, without maxillofacial abnormalities, were saved in DICOM format. The DICOM file was converted to STL (stereolithography) file in a 3D slicer software. Using CAD software (Rhinoceros Version 5.0 SR8, McNeel North America, Seattle, WA, USA), a model of an edentulous maxilla was constructed following the main anatomical characteristics of the patient’s bone: size, shape and absence of pathology. The command “ReduceMesh,” available as a plug-in for Rhinoceros, was used with 50% of relevance, allowing to smooth the structure with all normal faces oriented in the same direction [11]. The next step was to reconstruct the NURBS (non-uniform rational B-spline) surfaces from mesh or point cloud with specified precision. A 3D volumetric model of the bone was then finished based on the surface created by the curve network which was automatically generated. External hexagon implants (10 × 4.1 mm) were modeled with the external thread diameter being established according to the dimensions provided by the manufacturer (AS Technology Titanium Fix—São José dos Campos, Brazil). The platform had a diameter of 4.1 mm, similar to a regular conventional implant. The external hexagon was extruded (0.7 mm high) and attached to the previously created cylindrical body [12]. The minimum distance between the implants was 3 mm.

**Figure 2:**
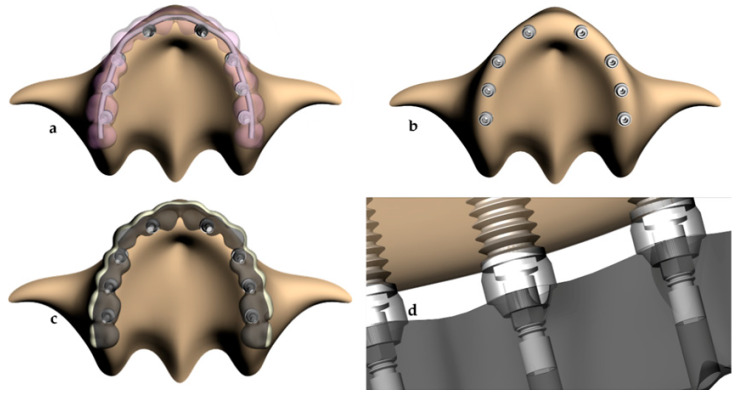
(**a**–**d**) Schematic illustration of the modeling based on the clinical parameters. (**a**) Conventional design based on a casted CoCr framework and acrylic resin. (**b**) The edentulous maxilla with the micro-conical abutments in position. (**c**) The prosthesis experimental design with a milled titanium framework with the lingual face of all teeth in metal, and the buccal face in resin. (**d**) Modeling of the intimate contact between abutment, screws, implants and framework.

A mini conical abutment was modeled for each implant and presented centralized insertion (2.5 × 4 mm). The abutment screw was modeled for each abutment, with a prosthetic screw on top of it [13]. Based on a generic maxillary arch, an IFDP was constructed in two different situations. The first situation with an IFDP supported by eight implants containing a CoCr framework with 2 mm^2^ of cross-section and a 4.1 mm coping screw for each abutment [13]. An acrylic resin prosthesis was modeled around the framework from the right second molar until the left second molar (Figure 2a).

The second design was the experimental treatment approach. The prosthesis was modeled with a titanium milled framework with the lingual face of all teeth in metal and resin at the buccal face (1 mm thickness) [13]. A mini-conical abutment was placed on each implant. The abutments presented centralized insertion with 2.5 × 4 mm. A 3D abutment screw was modeled for each abutment and a prosthetic screw on the top of it. The geometries of both prosthesis designs are presented in the Figure 2.

### 2.3. Material Poperties and Mechanical Loading

Each solid geometry was imported to the analysis software (ANSYS 17.2, ANSYS Inc., Houston, TX, USA) in STEP format. A 3D mesh was generated and tetrahedral elements were considered for the models. A convergence test of 10% [14] determined the total number of elements (440,225) and nodes (769,873) for the conventional design, as well as for the experimental design (424,264 elements and 743,712 nodes).

The Young’s modulus and Poisson ratio of each material/structure were assigned to each solid component with isotropic and homogeneous behavior (Table 1) [15,16,17,18].

With regards to the abutment screw, a pre-torque of 20 N·cm was used, whereas for the prosthetic screw, 10 N·cm was considered due to tightening corresponding to the torque performed in the clinic. The friction coefficient (μ) was set to 0.3 between all the metallic interfaces, 0.65 for the cortical bone-implant interface and 0.77 for the cancellous bone-implant interface [19,20,21]. The other contacts in the model were defined as perfectly bonded structures.

The boundary conditions were fixed in the x-, y- and z-directions on the bottom surfaces of cancellous bone to restrict unpredictable movement of the parts. The defined constrains allowed lateral deformation of the peri-implant bone [22]. On the external surface of the prosthesis at the buccal face (cingulum area) of the maxillary left canine, a load of 100 N was applied normal to the surface at 30°, mimicking a mandibular lateral movement [23].

Results were reported in terms of von Mises stress distribution for metal devices, microstrains (με) for bone tissue and displacements (mm) for the prosthesis [24].

## 3. Results

**Figure 3:**
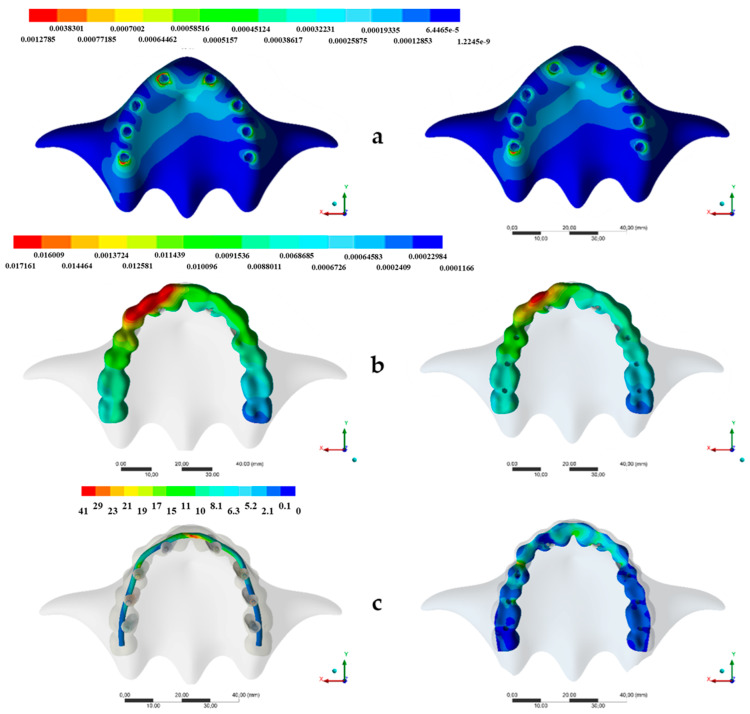
(**a**–**c**) Stress maps for the framework according to each design. Left: Conventional design based on a casted CoCr framework and acrylic resin. Right: The prosthesis experimental design. (**a**) Bone microstrain (µε), (**b**) Prosthesis displacement (mm) and (**c**) Von Mises stress distribution.

**Figure 4:**
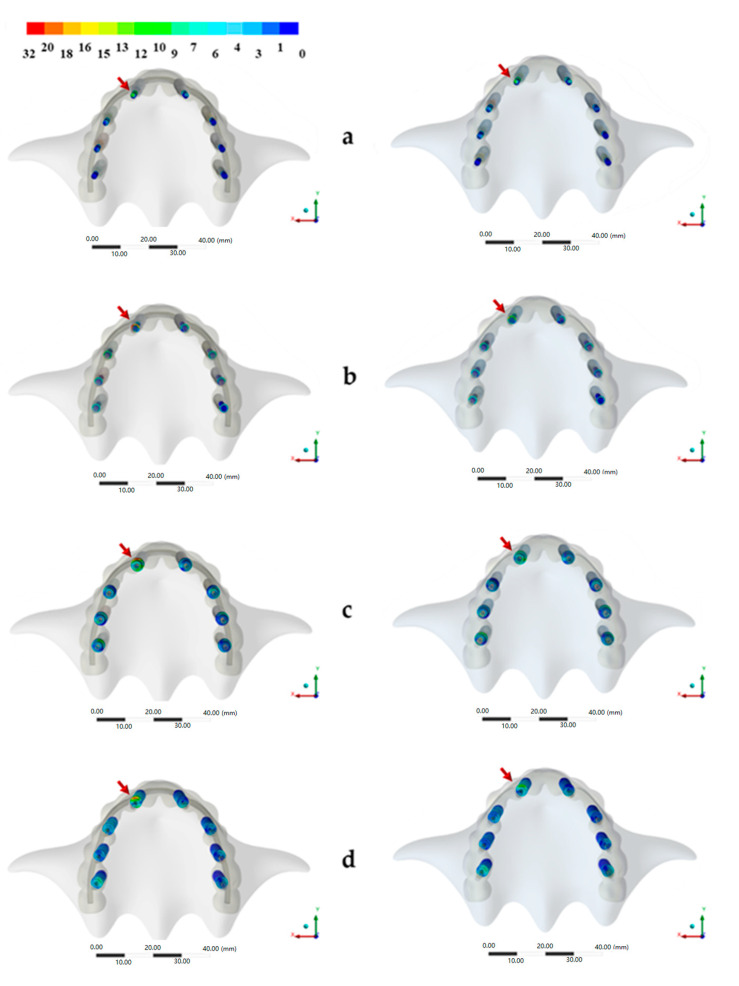
(**a**–**d**) Maps of von Mises stress distribution results in the conventional design (Left) and in the prosthesis experimental design (Right). (**a**) Prosthetic screw, (**b**) Abutment screw, (**c**) Abutment and (**d**) Implants. The red arrows indicate the areas with more concentrated stresses.

**Figure 5:**
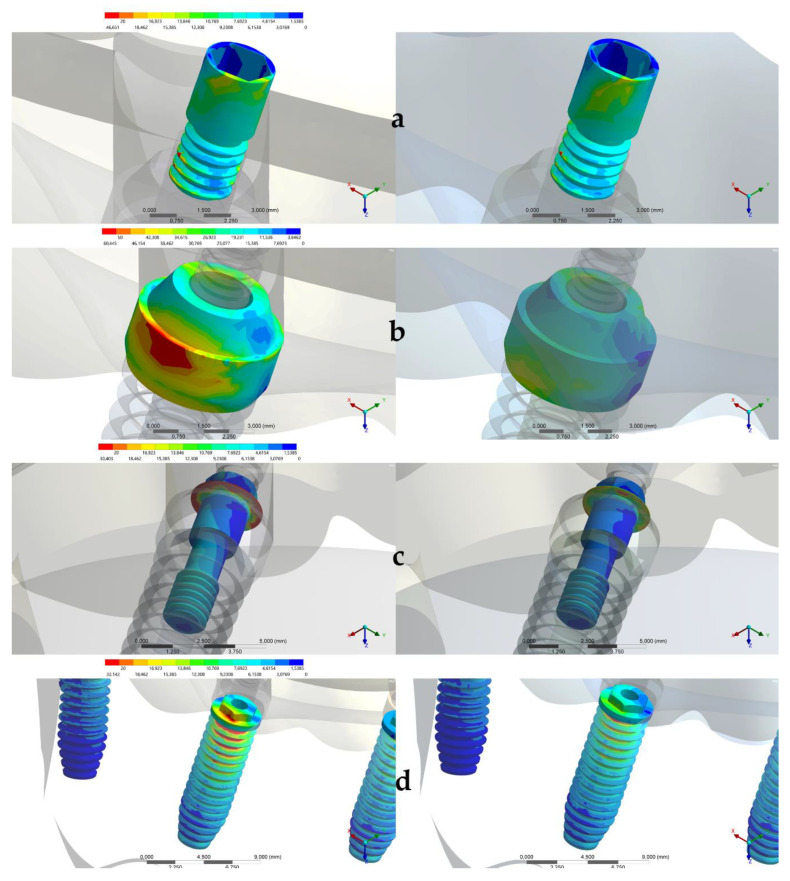
(**a**–**d**) An approximated view of the highest stress concentration maps in the conventional design (Left) and in the prosthesis experimental design (Right). (**a**) The prosthetic screw, (**b**) Abutment, (**c**) Abutment screw and (**d**) Implants.

The stress distribution in implant/prosthetic components and supporting tissues for all groups are displayed in Figure 3, Figure 4 and Figure 5.

The results in terms of displacement (mm) for prosthesis and stress peak values (MPa) in the framework, prosthetic screw, abutment screw, abutment and implant are summarized in Table 2.

All implants were able to distribute the load at the implant-bone interface. A more homogeneous distribution was noted in the case of experimental design, with fewer strain values observed for all peri-implant tissue (Figure 3). In any case, the obtained results in terms of strain values suggested no unwanted bone resorption and, hence, no mechanical problems.

The conventional group exhibited the highest stress values which were located in the prosthesis framework δ_max_ = 24 MPa) between the anterior implants (Figure 3), while the experimental design showed the lowest stress values (δ_max_ = 13.27) in the same region.

Figure 4 reports a map of stress distribution in all components and implants. The stress distribution in the prosthetic screw showed that the higher stress values were concentrated in the threads (Figure 5). The abutment with high stress concentration was the first (anterior) abutment on the same side of the applied load. The conventional design evidenced a greater possibility of damage if compared to the experimental one (Figure 5). For the abutment screw, the critical area was the head of the screw for both groups, regardless of the magnitude (Figure 5). For both designs, the stress was mainly concentrated in the implants, between the neck and the external hexagon region (Figure 5).

## 4. Discussion

This study evaluated the mechanical response of an alternative design for manufacturing an implant-supported full-arch dental prosthesis (IFDP) with limited occlusal vertical dimension. A similar treatment has been previously described in the literature, but there are no data regarding the improvements obtained using the proposed design [1].

The use of a theoretical study assists to explain why the experimental design could be useful. The null hypothesis was rejected due to the observed differences between both designs of structures in terms of stress concentration.

As reported in the literature, the use of a milled framework may be considered as a promising solution due to the smaller vertical mismatch, ensuring reduced misfit [5,24]. Several studies have used the finite element analysis (FEA) as a tool for the biomechanical evaluation of a full-arch implant prostheses [25,26,27]. Despite the limitations of a purely theoretical study, this methodology plays an important role in the hypothesis formulation. Thus, it enables to study and, hence, to avoid potential problems before they are even verified in the clinic [28,29]. Therefore, a preliminary theoretical analysis is advantageous for both the patient and the dentist, since it is not necessary to observe the prosthesis failure for the determination of the failure origin. Such a tool is able to calculate and predict mechanical problems. The accumulated stress in the implant-supported prosthesis infrastructure is directly related to the design and the employed material. It is well known that the generated stress may induce the material damage and failure [30].

In the current study, the proposed experimental design provided better results in terms of the biomechanical response, in comparison to the conventional one.

Another important factor is that this simulation was performed with eight implants supporting the prosthesis. The hypothesis that the presence of more implants would be beneficial to distribute the masticatory loads has been concisely reported in the literature [31,32]. Moreover, previous studies have demonstrated that there is a decrease in the strain generated in the peri-implant region when more implants are present [31,32]. It is important to note that the simulated implant number (eight) represents a condition that facilitates the masticatory load distribution, because it improves the model statics, making the entire system more rigid. Clinical conditions with lower implant number may present a different mechanical response with higher stress concentration [33]. Furthermore, for both treatments, values related to unwanted bone resorption or strain limit (>3000) were not observed [12,14]. The ultimate tensile strength for bone is related to the stress limit of 135 MPa [34] and for the titanium structures, it is related to the stress limit of 1035 MPa [35]. Considering the stress shielding problems, they could have been more evidenced in the experimental design prosthesis, since lower forces are distributed to the bone due to a more rigid simulated prosthesis. Therefore, the masticatory efficiency of the patient must be checked during the planning of this type of prosthesis to avoid bone resorption by disuse or reduced stimulus [36].

Although it seems obvious that a more rigid prosthesis possesses a higher strength, it is not desired that the fracture problem of the framework can be mitigated in exchange for other mechanical problems. For this reason, it was necessary to analyze each individual structure.

Therefore, some simplifications commonly found in theoretical studies that analyzed full-arch prostheses were avoided here. This is because, when many simplifications are considered, the analysis is performed faster using a shorter processing time, leading to results that may strongly differ from those obtained by using the detailed model [37]. However, the numerical validation of a simplified 3D model does not always allow to extrapolate all the needed results [14]. For example, in the literature there are studies on full-arch implant supported prosthesis that did not consider the prosthetic screws, abutment screws, acrylic resin, tooth anatomy, the study of screw pre-load non-linear contacts, or mesh convergent test [22,26,27,38]. The limitation of this study consisted in all implants presenting an ideal position, no defects or gaps between the mini-conical abutment and the prostheses, a perfectly symmetric maxilla and no saucerization of the peri-implant bone of any implant.

Taking into account that only under specific conditions these features and considerations do not significantly influence the results, the present research considered all of them in a theoretical model to reproduce the real situation as much as possible. However, an important limitation for this study is the fact that perfectly osseointegrated implants were considered in the maxilla. In addition, for different osseointegration levels, the bone implant behavior can be different and modify the present results [6].

In comparing the mechanical response of the models, it was possible to observe that the conventional design showed a higher stress concentration in the prosthetic framework. For the experimental design, the displacement of the prosthesis decreased, thus suggesting a reduced movement of the components and, consequently, lower stress values in the prosthetic screw, abutment and implant. The design of a prosthesis with a metallic lingual side was less damaging than the construction of a conventional prosthesis with a large quantity of acrylic resin. The authors agree that this simulation using a generic maxillary model and only one load application could not allow the results extrapolation for patients with different geometries, in term of stress magnitude. However, the superior mechanical response provided by the experimental design should be similar, regardless of the patient.

The abutment screw fracture in the case of mini-abutments for full-arch prosthesis is not commonly reported, differently from the framework fracture, which would theoretically be solved by manufacturing a prosthesis with the experimental design [2].

The experimental design can also be milled in zirconia [39]. The literature reports that the use of zirconia or metal for full-arch framework would achieve similar mechanical results for implants and peri-implant tissues, in addition to similar vertical fitting [5,22]. However, as a disadvantage, the constant friction of the zirconia framework with the abutments can increase the metal wear [40]. Depending on the prosthesis design, it also can facilitate wear of the antagonist arch [41]. In addition, the artificial tooth occlusal surface wear might be different between both designs, and the removal of the worn prosthesis can be difficult [41], requesting careful previous planning. A previous study reported that both prosthesis designs did not induced any bone damage due to the prosthesis weight and should be selected based in the masticatory load transmission [13,42]. Despite the limitations, the present study assists the clinician decision to select the experimental design when an improved mechanical response is needed [43].

## 5. Conclusions

Although an alternative design of a stronger framework with less stress concentration was proposed in the present study, its major disadvantages could be related to the potentially higher laboratory costs and aesthetics.

Benefiting from the obtained results according to this experiment conditions, the following conclusions were drawn:

1. An alternative design was proposed to manufacture a stronger framework;
2. Even though strain values related to unwanted bone resorption were not observed for both designs, the experimental prosthetic design with customized milled framework resulted in lower stress concentration and, hence, in lower possibility of damage, if compared to the conventional one;
3. The current study would represent an important step in the design and analysis of an implant-supported full-arch dental prosthesis with limited occlusal vertical dimension in terms of mechanical improvements.

## Figures and Tables

**Table 1:** Mechanical properties of the materials/structures used in the current study.

Material/Structure	Young’s Modulus (GPa)	Poisson Ratio
Titanium	110	0.35
CoCr	220	0.30
Cancellous bone	1.37	0.30
Cortical bone	13.7	0.30
Acrylic Resin	2.7	0.35

**Table 2:** Results in terms of bone microstrain (µε), prosthesis displacement (mm) and stress peak values (MPa) according to the framework.

Variables	Conventional Design	Experimental Design
Bone microstrain	1420	1260
Prosthesis displacement	0.023	0.017
Stress on the framework	24.31	13.27
Stress on the prosthetic screw	14.15	12.23
Stress on the abutment	23.23	13.17
Stress on the abutment screw	24.36	25.42
Stress on the implant	28.12	20.72
